# Febrifuge and Antiperiodic Properties of Chloroform

**Published:** 1850-09

**Authors:** 


*Febrifuge and Antiperiodic Properties of Chloroform.*—M. Delioux lately read to the French Academy of Medicine, a communication on this subject, in which he spoke of the benefit he had derived from the internal use of chloroform in intermittent fevers which had resisted quinine. It would appear from the tenor of the author's remarks, that no unpleasant effects have been witnessed, the only action of the medicine being that of suspending the febrile paroxysms. The usual dose is from thirty to forty drops, given in three portions a few hours before the expected access, taking care that the last dose is given about three hours prior to the fit. In obstinate tertian and quartan fevers the author gives the medicine daily, increasing the dose on the days of the access, and subsequently reducing it.—*New York Medical Gazette.*

